# Long Feeding High-Fat Diet Induces Hypothalamic Oxidative Stress and Inflammation, and Prolonged Hypothalamic AMPK Activation in Rat Animal Model

**DOI:** 10.3389/fphys.2018.00818

**Published:** 2018-07-06

**Authors:** Gina Cavaliere, Emanuela Viggiano, Giovanna Trinchese, Chiara De Filippo, Antonietta Messina, Vincenzo Monda, Anna Valenzano, Raffaele I. Cincione, Christian Zammit, Fabiano Cimmino, Angela Catapano, Francesco Sessa, Giovanni Messina, Marcellino Monda, Marianna Crispino, Maria Pina Mollica

**Affiliations:** ^1^Department of Biology, University of Naples Federico II, Naples, Italy; ^2^Section of Human Physiology, Department of Experimental Medicine, Università degli Studi della Campania “Luigi Vanvitelli”, Naples, Italy; ^3^Prenatal Medicine, ULSS6 Euganea, Padua, Italy; ^4^Unit of Dietetics and Sports Medicine, Section of Human Physiology, Department of Experimental Medicine, Università degli Studi della Campania “Luigi Vanvitelli”, Naples, Italy; ^5^Department of Clinical and Experimental Medicine, University of Foggia, Foggia, Italy; ^6^Department of Anatomy, University of Malta, Msida, Malta

**Keywords:** age, obesity, hypothalamic oxidative stress, inflammation, AMPK activation

## Abstract

**Scope:** The hypothalamus is a key brain region involved in the control of feeding and energy expenditure. Hypothalamic inflammation and oxidative stress are landmarks of both obesity and aging processes, although the molecular mechanisms are still unknown. Therefore, with the aim to understand the neurobiological mechanisms of energy homeostasis during aging, we evaluate the effects of long feeding high-fat diet (HFD) in rats, at different age, on modulation of hypothalamic molecular pathway, oxidative stress, and inflammation.

**Procedures:** Male Wistar rats were divided into two groups: control group, receiving standard diet (CD), and treated group, receiving HFD. Both groups were treated with the appropriate diet for 1, 3, 6, 12, or 18 weeks. We investigated energy balance and body composition, as well as lipid profile, homeostatic model assessment index, and inflammatory state in serum. Furthermore, we also analyzed, at hypothalamic level, inflammation and oxidative stress, and adenosine monophosphate-dependent kinase (AMPK) and pAMPK expression levels.

**Results:** Our data showed that aging and HFD induce increased energy intake and energy efficiency and decreased energy expenditure associated, at hypothalamic level, with inflammation and oxidative stress and activation of AMPK.

**Conclusion:** Our results indicate that the age at which HFD feeding starts and the diet duration are critical in obesity development. The prolonged activation of hypothalamic AMPK may be related to the alterations in energy homeostasis.

## Introduction

The prevalence of obesity is progressively increasing worldwide and is reaching epidemic proportions. This alarming increase is mainly related to sedentary lifestyle and high consumption of fat-rich food. Weight gain depends on the imbalance between food intake and energy expenditure, and both parameters are tightly controlled by the hypothalamus (De Souza et al., 2005). Under physiological conditions, anabolic and catabolic processes are carried out continuously in the body. However, the relative activity of these two processes varies between different physiological situations, as for instance during aging. In the initial stages of development, the anabolic mechanisms exceed the catabolic ones to support the growth. During adulthood, the same positive ratio between anabolic and catabolic processes may lead to overweight, since the extra calories are mostly stored as fat. Therefore, overweight is frequently observed in middle-aged subjects (age-related obesity). In the elderly, the catabolic processes often increase, leading to weight and muscle mass loss (age-related anorexia) (Pétervári et al., 2011). The progressive increase in adiposity occurring throughout most of adulthood due to the impairment of mechanisms that normally control energy homeostasis is a normal aging process (Iossa et al., 2002, 2003; Xu et al., 2005). It is well known that the incidence of obesity increases in middle-aged humans, and it may accelerate the aging processes (Balaskó et al., 2013), thereby making aging and obesity tightly interconnected. Aged rats were reported to develop leptin resistance that is typically associated with obesity (Scarpace et al., 2000). It has also been demonstrated that both the consumption of a high-fat diet (HFD) and the aging process evoke an inflammatory response in the hypothalamus, inducing resistance to insulin and leptin. These hypothalamic malfunctions result in a defective control of food intake and energy expenditure, leading to hyperphagia, obesity, and type 2 diabetes (Cavadas et al., 2016). Thus, hypothalamic inflammation has been identified as a crucial step not only in the development of obesity but also in the aging processes, although the molecular mechanisms underlying the inflammatory response of hypothalamic neurons to obesity and aging is still partially unknown.

One important molecule counteracting inflammatory signaling pathways is adenosine monophosphate-dependent kinase (AMPK) (Hernández-Aguilera et al., 2013). AMPK, a serine/threonine kinase, plays a crucial role in detecting the energy status of most eukaryotic cells, measuring the ratio AMP/ATP and ADP/ATP. This ratio increases when cellular energy falls (Hardie et al., 2012). AMPK is activated into pAMPK by metabolic stresses, and acts to restore energy homeostasis, thereby increasing energy production and restoring energy waste (Hardie, 2014). Given its involvement in managing cellular energy resources, AMPK plays a leading role in human metabolism. It is also involved in several pathological conditions, such as diabetes and heart disease, Alzheimer’s disease, and immune responses (Martínez de Morentin et al., 2016). In the central nervous system, the AMPK pathway integrates hormonal signals with neuronal networks, thereby playing a key role in regulating energy homeostasis (López, 2017). In particular, the hypothalamic AMPK activates specific metabolic pathways, to modulate feeding and energy expenditure, muscle metabolism, hepatic function, and glucose homeostasis.

In this study, we analyzed the effects of long feeding HFD in Wistar male rats at different age. Rats kept in laboratory represent a very useful tool to study obesity-related diseases, since they display a sedentary behavior, due to standard stabulation conditions. Therefore, using this animal model, we performed experiments, at different age, to link long-term high-fat feeding and aging with modulation of the hypothalamic molecular pathway, oxidative stress, and inflammation.

## Materials and Methods

All chemicals were purchased from Sigma-Aldrich (St. Louis, MO, United States), unless otherwise specified.

Young male Wistar rats at 60 days of age and 345 ± 7 g as average body weight were individually caged in a temperature-controlled room and exposed to a daily 12/12 h light/dark cycle with free access to chow diet and drinking water. Rats were divided into two experimental groups according to a different dietary regimen: the first group (control diet, CD) received a standard diet (10.6% fat J/J, 29% protein J/J, 60.4% carbohydrate J/J); the second group (HFD) received HFD (40% fat J/J, 29% protein J/J, 31% carbohydrate J/J). The animals from both groups were treated with the appropriate diet for 1, 3, 6, 12, or 18 weeks (*n* = 6 for each group and time point). An additional group (*n* = 6) was sacrificed at the beginning of the study to establish baseline measurements of body compositions.

At the end of the experimental treatments, the rats were anesthetized by i.p. injection of chloral hydrate (40 mg/100 g body weight), decapitated with a guillotine, and the blood was taken from the inferior cava vein. The hypothalamus was quickly dissected from the brain and transferred in the appropriate buffer. All the samples that were not immediately used were stored at -80°C.

### Measurement of Oxygen Consumption, Carbon dioxide Production, and Respiratory Quotient

Following an adaption period to the experimental environment (at least 1 day), oxygen consumption (VO_2_) and carbon dioxide production (VCO_2_) were recorded by a monitoring system (Panlab s.r.l., Cornella, Barcelona, Spain) that is composed of a four-chambered indirect open-circuit calorimeter, designed for continuous and simultaneous monitoring. VO_2_ and VCO_2_ were measured every 15 min (for 3 min) in each chamber for a total of 6 h (from 8:00 am to 14:00 pm). The mean VO_2_, VCO_2_, and respiratory quotient (RQ) values were calculated by the “Metabolism H” software (Dominguez et al., 2009).

### Energy Balance

During the experimental period, the body weight and food intake were monitored daily to calculate weight gain and gross energy intake. Food spilled was carefully collected and accounted for in the food intake calculations. Feces and urine were also collected daily, dried, and ground to a powder before determining their energy content with a bomb calorimeter calibrated with dry benzoic acid standard (Parr adiabatic calorimeter; Parr Instruments Co., Moline, IL, United States).

The gross energy content of the diets was also determined with the bomb calorimeter, and the values were 15.88 kJ/g for the standard diet and 20.0 kJ/g for HFD.

Metabolizable energy (ME) intake was determined by subtracting the energy measured in feces and urine from the gross energy intake, which was determined from the daily food consumption and gross energy density.

Energy balance assessments were conducted by the comparative carcass evaluation (Mollica et al., 2017). Briefly carcasses were weighed, autoclaved for 90 min, chopped into small pieces, thoroughly mixed, and homogenized with a mass of water equal to twice the carcass weight in a Polytron homogenizer (Polytron Kinematica AG, Littau/Lucerne, Switzerland). Aliquots of the homogenates were desiccated at 70°C in a vacuum oven. Small pellets (about 200 mg) of the dried homogenate were made and the body energy content was measured with the bomb calorimeter (Parr Instruments Co.). Energy efficiency was calculated as the percentage of body energy retained per ME intake, and energy expenditure was determined as the difference between ME intake and energy gain.

### Body Composition Measurements

Aliquots of the carcass homogenate were analyzed for lipid, protein, and water content. Water content was determined by the difference in weight of the homogenate before and after drying at 70°C in a vacuum oven. Lipid content was determined gravimetrically after extraction in chloroform/ methanol and evaporation to constant weight by a rotating evaporator (Heidolph, Kelheim, Germany) by the method of Folch et al. (1957). The energy as lipid was calculated from the lipid content by using the coefficient of 39.2 kJ/g for the energy content of lipid. Protein content was determined by the Biuret method after extraction in sodium dodecyl sulfate ± NaOH as described by Brooks et al. (1995). The energy as protein was calculated from the protein content by using the value of 23.5 kJ/g for the energy content of protein.

### Serum Parameters

The serum levels of cholesterol, triglycerides, NEFA, and glucose were measured with standard procedures. The serum levels of insulin (Mercodia AB, Uppsala, Sweden), TNF-α, IL1β, and IL6 (Biovendor R&D, Brno, Czechia), and adiponectin and leptin (B-Bridge International Mountain View, CA, United States) were measured using commercially available ELISA kits.

### Hypothalamic Parameters

To determine the lipid peroxidation in hypothalamic homogenate, the level of malondialdehyde (MDA) was measured using the thiobarbituric acid reaction (TBAR) method. MDA reacts with thiobarbituric acid (TBA) to form a pink chromogen that is detected at the wavelength of 532 nm. MDA values were expressed as nanomoles per milligram of protein (Lu et al., 2009). The levels of TNF-α, IL1β, and IL6 in the hypothalamic homogenate was determined as previously described (Spagnuolo et al., 2015).

### Redox Status and Nuclear Factor Erythroid 2-Related Factor (Nrf2) Activated Enzymes Activities

Reduced glutathione (γ-L-glutamyl-L-cysteinyl-glycine, GSH) and oxidized glutathione (γ-glutamyl-L-cysteinyl glycine disulfide, GSSG) concentrations in the hypothalamus were measured using the dithionitrobenzoic acid (DTNB) GSSG reductase recycling assay (Bergamo et al., 2007). The GSH/GSSG ratio was used as an oxidative stress marker. The enzymatic activities of glutathione *S*-transferase (GST) and NAD(P)H–quinone oxidoreductase (NQO1) were evaluated spectrophotometrically in cytoplasmic extracts with standard protocols (Benson et al., 1980; Levine et al., 1990).

### ROS Assay

The levels of ROS were determined as previously reported (Montoliu et al., 1994). An appropriate volume of tissue homogenate was diluted in 100 mM potassium phosphate buffer (pH 7.4) and incubated in a final concentration of 5 μM dichlorofluorescein diacetate (Sigma-Aldrich) in dimethyl sulfoxide for 15 min at 37°C. The dye-loaded samples were centrifuged at 12,500 × *g* per 10 min at 4°C. The pellet was resuspended in 5 ml of 100 mM potassium phosphate buffer (pH 7.4) at 4°C, and incubated for 60 min at 37°C. The fluorescence measurements were performed at 488 nm for excitation and 525 nm for emission wavelengths. ROS were quantified using dichlorofluorescein standard curve in dimethyl sulfoxide (0–1 mM).

### CuZn–SOD Activity

CuZn–SOD activity was determined using spectrophotometer at 550 nm, 25°C. The assay for CuZn–SOD was based on its ability to inhibit the oxidation of oxymine by the xanthine–xanthine oxidase system (Oyanagui, 1984). CuZn–SOD levels were expressed as units per mg protein.

### Western Blot

The hypothalamus was homogenized in the lysis buffer (10 mM HEPES, 10 mM KCl, 1.5 mM MgCl_2_, 12% glycerol, 0.5 mM DTT, 0.1 mM EGTA) with a cocktail of protease inhibitors (Sigma-Aldrich). Separation of proteins and Western blot analyses were performed as previously described (Chun et al., 2004). Briefly, proteins (20 or 40 μg/lane) were separated on 12% SDS-PAGE and transferred to nitrocellulose membranes. The blots were incubated with AMPKα rabbit monoclonal antibody (Cell Signaling Technology; 1:1000), pAMPKα (Thr172) rabbit monoclonal antibody (Cell Signaling Technology; 1:1000), and α-tubulin mouse antibody (Sigma-Aldrich; 1:1000) overnight at 4°C and then with secondary antibody against rabbit or mouse IgG (Promega; 1:2500) for 1 h at RT. The signals were visualized with the ECL system (Pierce). The expression level of α-tubulin was used to normalize the data.

### Statistical Analysis

Statistical analyses were carried out using GraphPad Prism (GraphPad Software, San Diego, CA, United States). Two-way ANOVA considering the factor diet (CD or HFD) and age (1, 3, 6, 12, or 18 weeks) followed by Bonferroni’s *post hoc* test was used to evaluate differences between the groups for all the parameters analyzed. Student’s *t*-test was used to evaluate the difference in AMPK or pAMPK protein expression in the hypothalamus between treated and control group at each time of treatment. *p*-Values smaller than 0.05 were considered statistically significant.

### Ethics Statement

Procedures involving animals and their care were conducted in conformity with international and national law and policies (EU Directive 2010/63/EU for animal experiments, ARRIVE guidelines and the Basel declaration including the 3R concept). The procedures reported here were approved by the Institutional Committee on the Ethics of Animal Experiments (CSV) of the University of Naples Federico II and by the Ministero della Salute.

## Results

### Aging and HFD Increase Body Weight Gain and ME Intake

The results of two-way ANOVA analysis for body weight showed significant effect for the age [*F*(4,50) = 569.4; *p* < 0.01], diet [*F*(1,50) = 441.8; *p* < 0.01], and the interaction between the age and diet [*F*(4,50) = 51.30; *p* < 0.01].

The results of two-way ANOVA analysis for body lipid percentage showed significant effect for the age [*F*(4,50) = 133.8; *p* < 0.01], diet [*F*(1,50) = 60.27; *p* < 0.01], and the interaction between the age and diet [*F*(4,50) = 9.814; *p* < 0.01].

The results of two-way ANOVA analysis for body energy content showed significant effect for the age [*F*(4,50) = 262.7; *p* < 0.01], diet [*F*(1,50) = 85.31; *p* < 0.01], and the interaction between the age and diet [*F*(4,50) = 13.97; *p* < 0.01].

**FIGURE 1:**
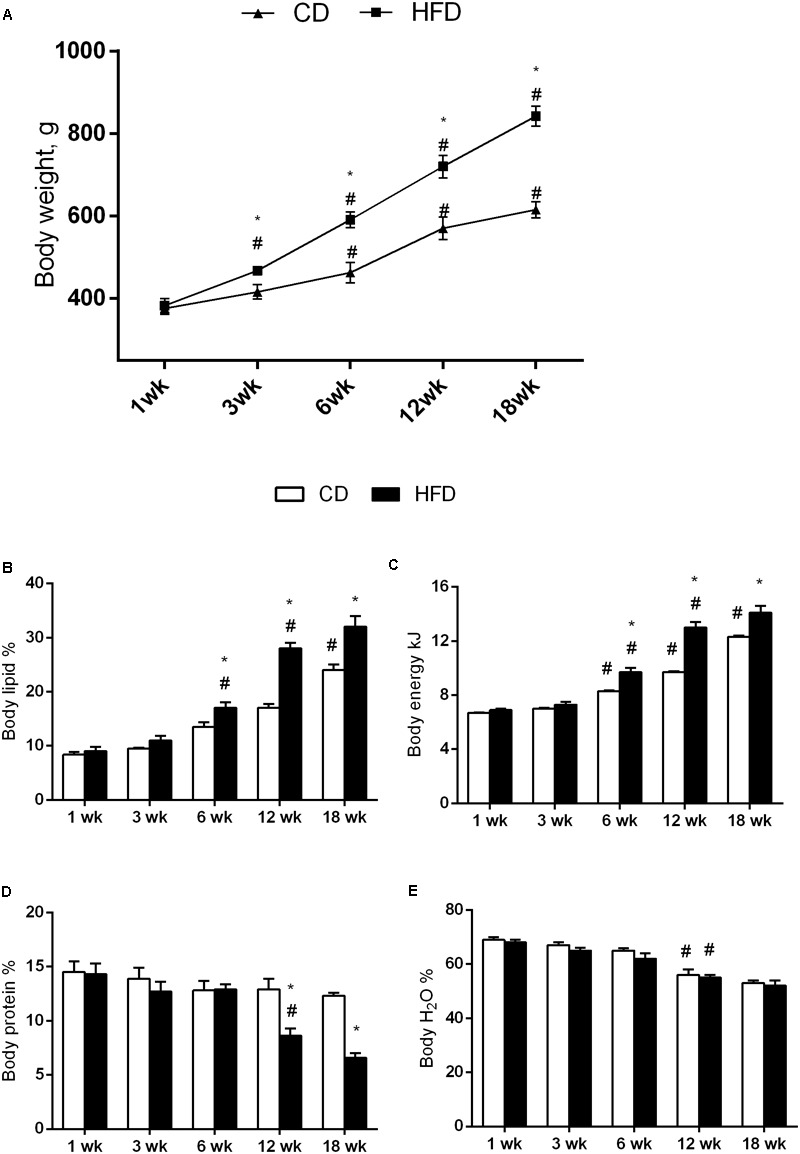
Effects of HFD and age on body weight and composition. All the parameters were measured throughout the experimental period (1–18 weeks). Body weight **(A)**, body lipid **(B)**, body energy **(C)**, body protein **(D)**, and body water **(E)** are shown. Data are indicated as means ± SEM from *n* = 6 animals/group. ^#^Significantly different compared to previous age, *p* < 0.05; ^∗^significantly different compared to CD group, *p* < 0.05.

In both HFD and CD groups, the body weight, body lipid, and body energy content progressively increased with age. All parameters were higher in HFD than in CD group at each time point, but the difference was statistically significant only after 6 weeks of treatment (**Figures 1A–C**).

The results of two-way ANOVA analysis for body protein percentage showed significant effect for the age [*F*(4,50) = 12.20; *p* < 0.01], diet [*F*(1,50) = 19.32; *p* < 0.01], and the interaction between the age and diet [*F*(4,50) = 5.093; *p* < 0.01].

Body protein did not change with age in CD group, while in the HFD group, at 12 and 18 weeks, decreased significantly when compared with the other time points. In addition, this parameter in the HFD group at 12 and 18 weeks was significantly lower than in CD group of the same age (**Figure 1D**).

The results of two-way ANOVA analysis for body water percentage showed significant effect for the age [*F*(4,50) = 50.45; *p* < 0.01], while the diet [*F*(1,50) = 3.402; *p* > 0.05] and the interaction between the age and diet was not significant [*F*(4,50) = 0.2127; *p* > 0.05].

Body water decreased with age in both the HFD and CD groups, although the differences were significant only at 12 and 18 weeks compared to the other age groups (**Figure 1E**).

The results of two-way ANOVA analysis for body weight gain showed significant effect for the age [*F*(4,50) = 122,251; *p* < 0.01], diet [*F*(1,50) = 79,384; *p* < 0.01], and the interaction between the age and diet [*F*(4,50) = 12,448; *p* < 0.01].

**FIGURE 2:**
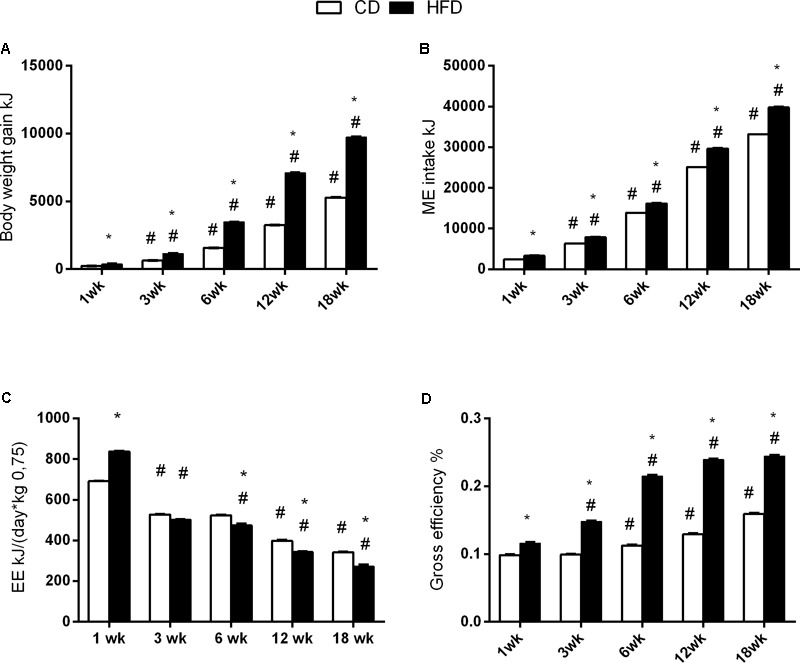
Effects of HFD and age on energy balance. All the parameters were measured throughout the experimental period (1–18 weeks). Body weight gain **(A)**, ME intake **(B)**, energy expenditure **(C)**, and efficiency **(D)** are shown. Data are indicated as means ± SEM from *n* = 6 animals/group. ^#^Significantly different compared to previous age, *p* < 0.05; ^∗^significantly different compared to CD group, *p* < 0.05.

The body weight gain significantly increased with age at each time points and was higher in the HFD when compared to the CD group (**Figure 2A**).

The results of two-way ANOVA analysis for ME intake showed significant effect for the age [*F*(4,50) = 90,262; *p* < 0.01], diet [*F*(1,50) = 5685; *p* < 0.01], and the interaction between the age and diet [*F*(4,50) = 642.6; *p* < 0.01].

The ME intake (kJ) was significantly higher in the HFD group compared to the CD group at every time point (**Figure 2B**).

The results of two-way ANOVA analysis for energy expenditure showed significant effect for the age [*F*(4,50) = 2008; *p* < 0.01], diet [*F*(1,50) = 9.017; *p* < 0.01], and interaction between the age and diet [*F*(4,50) = 126.7; *p* < 0.01].

The energy expenditure progressively decreased with age in both groups, and significantly decreased in the HFD group, when compared to CD from 6 weeks onward (**Figure 2C**).

The results of two-way ANOVA analysis for gross efficiency showed significant effect for the age [*F*(4,50) = 3201; *p* < 0.01], diet [*F*(1,50) = 13,322; *p* < 0.01], and the interaction between the age an d diet [*F*(4,50) = 755.5; *p* < 0.01].

The gross efficiency increased with age starting at 6 weeks for the CD group, and at 3 weeks for the HFD group. In addition, the gross efficiency in the HFD group was significantly higher than in CD group at each time point (**Figure 2D**).

### Aging and HFD Reduced VO_2_ and VCO_2_

The results of two-way ANOVA analysis for VO_2_ showed significant effect for the age [*F*(4,50) = 190.0; *p* < 0.01], diet [*F*(1,50) = 69.90; *p* < 0.01], and the interaction between the age and diet [*F*(4,50) = 3.533; *p* < 0.01].

The results of two-way ANOVA analysis for VCO_2_ showed significant effect for the age [*F*(4,50) = 111.1; *p* < 0.01], diet [*F*(1,50) = 58.16; *p* < 0.01], and the interaction between the age and diet [*F*(4,50) = 4.869; *p* < 0.01].

The results of two-way ANOVA analysis for RQ showed significant effect for the diet [*F*(1,50) = 289.6; *p* < 0.01], age [*F*(4,50) = 6.416; *p* < 0.01], and the interaction between the age and diet [*F*(4,50) = 6.217; *p* < 0.01].

**FIGURE 3:**
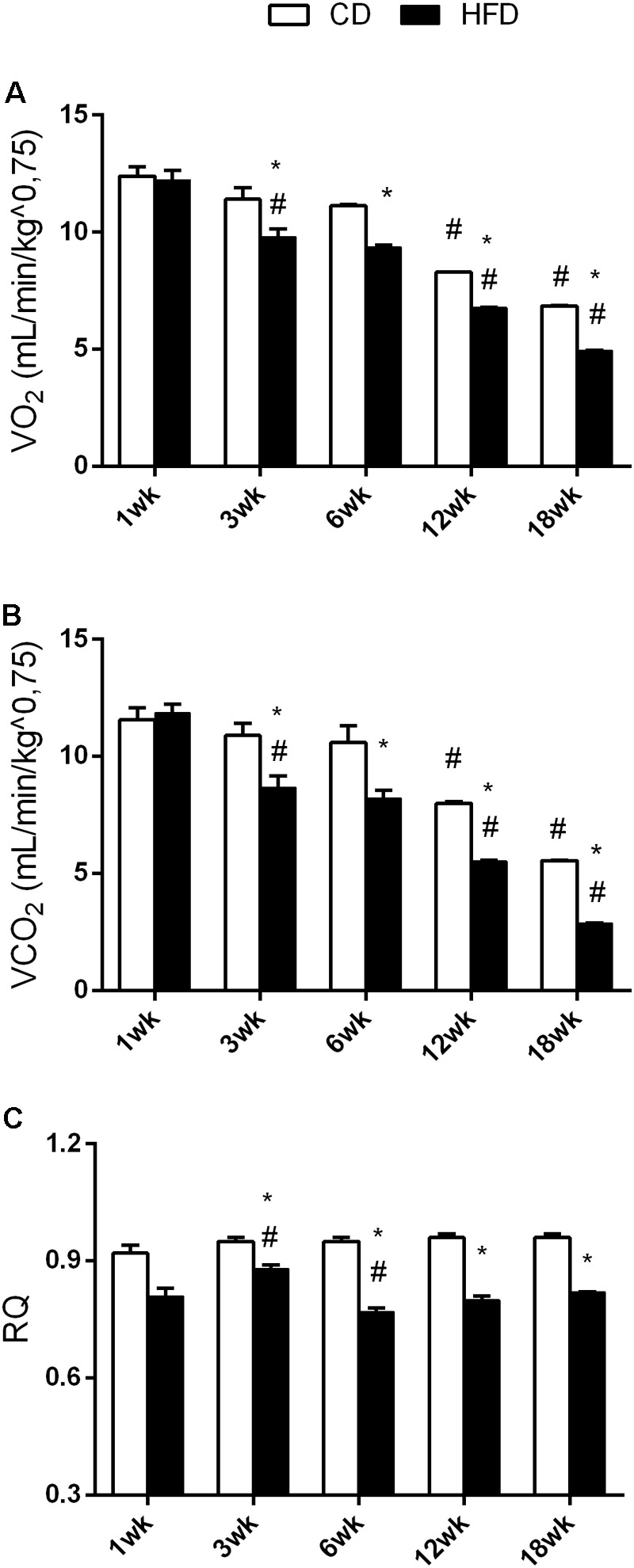
Effects of HFD and age on VO_2_, VCO_2_, and RQ. All the parameters were measured throughout the experimental period (1–18 weeks). VO_2_ **(A)**, VCO_2_ **(B)**, and RQ **(C)** are shown. Data are indicated as means ± SEM from *n* = 6 animals/group. ^#^Significantly different compared to previous age, *p* < 0.05; ^∗^significantly different compared to CD group, *p* < 0.05.

In the CD group, VO_2_ and VCO_2_ values at 12 and 18 weeks were significantly lower than in the younger cohort (**Figures 3A,B**) while no change was observed in the RQ value (**Figure 3C**). In the HFD group, VO_2_ and VCO_2_ values significantly decreased with age (**Figures 3A,B**). RQ values at 6, 12, and 18 weeks were significantly lower than at 1 and 3 weeks (**Figure 3C**). Moreover, VO_2_, VCO_2_, and RQ significantly decreased in the HFD group, when compared to the CD from 3 weeks onward.

### Aging and HFD Modulate Plasma Lipids, Hormones, and TNF-α

The results of two-way ANOVA analysis for TG levels in the plasma showed significant effect for the age [*F*(4,50) = 21.25; *p* < 0.01], diet [*F*(1,50) = 90.66; *p* < 0.01], and the interaction between the age and diet [*F*(4,50) = 5.592; *p* < 0.01].

**FIGURE 4:**
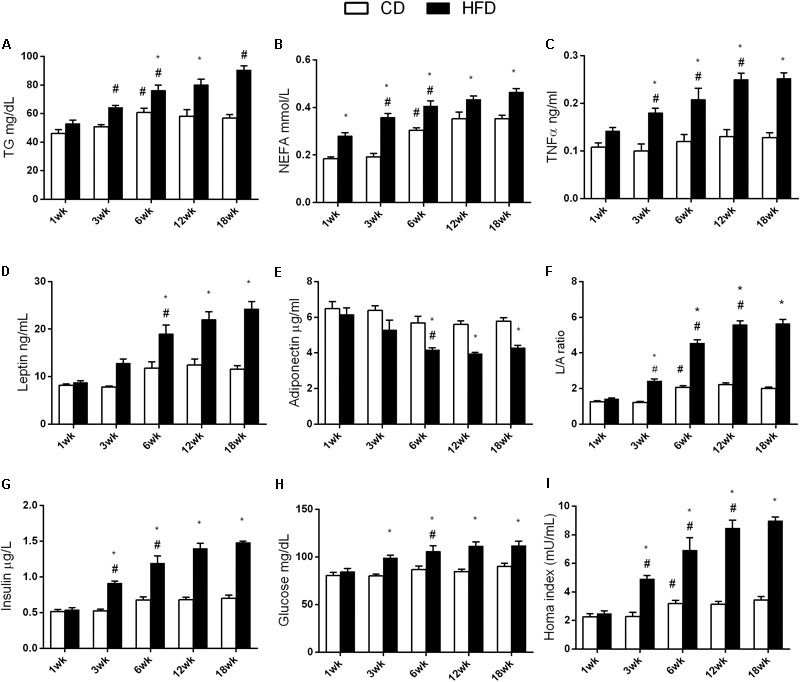
Effects of HFD and age on serum metabolic parameters and proinflammatory markers. All the parameters were measured throughout the experimental period (1–18 weeks). Triglycerides **(A)**, NEFA **(B)**, TNF-α **(C)**, leptin **(D)**, adiponectin **(E)**, L/A ratio **(F)**, insulin **(G)**, glucose **(H)**, and HOMA index **(I)** are shown. Data are indicated as means ± SEM from *n* = 6 animals/group. ^#^Significantly different compared to previous age, *p* < 0.05; ^∗^significantly different compared to CD group, *p* < 0.05.

TG values were higher at 6, 12, and 18 weeks than at 1 and 3 weeks in the CD group while, in the HFD group, these values increased progressively with age. In the HFD group, TG values significantly increased compared to the CD group, starting at 6 weeks of treatment (**Figure 4A**).

The results of two-way ANOVA analysis for NEFA levels in the plasma showed significant effect for the age [*F*(4,50) = 43.37; *p* < 0.01] and diet [*F*(1,50) = 116.1; *p* < 0.01], while the interaction between the age and diet was not significant [*F*(4,50) = 1.943; *p* > 0.05].

NEFA values were higher at 6, 12, and 18 weeks, when compared to the values taken at 1 and 3 weeks, in both the HFD and the CD groups. In the HFD group, NEFA values were significantly higher compared to those in the CD group at each time point (**Figure 4B**).

The results of two-way ANOVA analysis for TNF-α levels in the plasma showed significant effect for the age [*F*(4,50) = 9.041; *p* < 0.01], diet [*F*(1,50) = 105.0; *p* < 0.01], and the interaction between the age and diet [*F*(4,50) = 3.493; *p* < 0.01].

TNF-α did not change with age in the CD group, while in the HFD group, it increased significantly with age, starting at 3 weeks. This increase was significantly different from the corresponding value in the CD group, from 3 weeks onward (**Figure 4C**).

The results of two-way ANOVA analysis for leptin levels in the plasma showed significant effect for the age [*F*(4,50) = 26.05; *p* < 0.01], diet [*F*(1,50) = 88.39; *p* < 0.01], and the interaction between the age and diet [*F*(4,50) = 7.58; *p* < 0.01].

The results of two-way ANOVA analysis for adiponectin levels in the plasma showed significant effect for the age [*F*(4,50) = 9.465; *p* < 0.01] and diet [*F*(1,50) = 39.24; *p* < 0.01], while the interaction between the age and diet was not significant [*F*(4,50) = 1.559; *p* > 0.05].

Leptin and adiponectin did not change with age in the CD group. In the HFD group, these parameters were significantly different at 6, 12, and 18 weeks when compared to the values taken at 1 and 3 weeks, and also from the values taken at the corresponding age in the CD group (**Figures 4D,E**).

The results of two-way ANOVA analysis for leptin/adiponectin (L/A) ratio showed significant effect for the age [*F*(4,50) = 164.4; *p* < 0.01], diet [*F*(1,50) = 686.8; *p* < 0.01], and the interaction between the age and diet [*F*(4,50) = 63.65; *p* < 0.01].

The leptin/adiponectin ratio was higher at 6, 12, and 18 weeks, than at 1 and 3 weeks in the CD group. In the HFD group, this ratio increased progressively with age and was higher than that in the CD group (**Figure 4F**).

The results of two-way ANOVA analysis for insulin levels in the plasma showed significant effect for the age [*F*(4,50) = 43.13; *p* < 0.01], diet [*F*(1,50) = 229.9; *p* < 0.01], and the interaction between the age and diet [*F*(4,50) = 17.80; *p* < 0.01].

Insulin did not change with age in the CD group, while in the HFD group, it increased significantly with age, starting at 3 weeks. It was also significantly different from the corresponding age in the CD group, from 3 weeks onward (**Figure 4G**).

The results of two-way ANOVA analysis for glucose levels in the plasma showed significant effect for the age [*F*(4,50) = 7.604; *p* < 0.01] and diet [*F*(1,50) = 55.94; *p* < 0.01], while the interaction between the age and diet was not significant [*F*(4,50) = 2.421; *p* > 0.05].

Glucose level did not change with age in the CD group. In the HFD group, glucose level was significantly different at 6, 12, and 18 weeks, when compared with values taken at from 1 and 3 weeks and was also significantly different from the corresponding age in the CD group, starting at 3 weeks (**Figure 4H**).

The results of two-way ANOVA analysis for homeostatic model assessment (HOMA) index showed significant effect for the age [*F*(4,50) = 32.29; *p* < 0.01], diet [*F*(1,50) = 192.9; *p* < 0.01], and the interaction between the age and diet [*F*(4,50) = 15.13; *p* < 0.01].

The HOMA index increased at 6, 12, and 18 weeks, when compared to 1 and 3 weeks in the CD group. In the HFD group, the index increased progressively with age and was significantly different from the corresponding age in the CD group, starting at 3 weeks (**Figure 4I**).

### Aging and HFD Modulate Oxidative Stress in the Brain

The results of two-way ANOVA analysis for MDA production showed significant effect for the age [*F*(4,50) = 124.8; *p* < 0.01], diet [*F*(1,50) = 140.6; *p* < 0.01], and the interaction between the age and diet [*F*(4,50) = 5.785; *p* < 0.01].

The results of two-way ANOVA analysis for ROS production showed significant effect for the age [*F*(4,50) = 64.96; *p* < 0.01], diet [*F*(1,50) = 104.6; *p* < 0.01], and the interaction between the age and diet [*F*(4,50) = 4.878; *p* < 0.01].

The results of two-way ANOVA analysis for TNF-α levels showed significant effect for the age [*F*(4,50) = 84.50; *p* < 0.01], diet [*F*(1,50) = 175.7; *p* < 0.01], and the interaction between the age and diet [*F*(4,50) = 10.72; *p* < 0.01].

The results of two-way ANOVA analysis for IL1β levels showed significant effect for the age [*F*(4,50) = 32,227; *p* < 0.01], diet [*F*(1,50) = 125,952; *p* < 0.01], and the interaction between the age and diet [*F*(4,50) = 11,529; *p* < 0.01].

**FIGURE 5:**
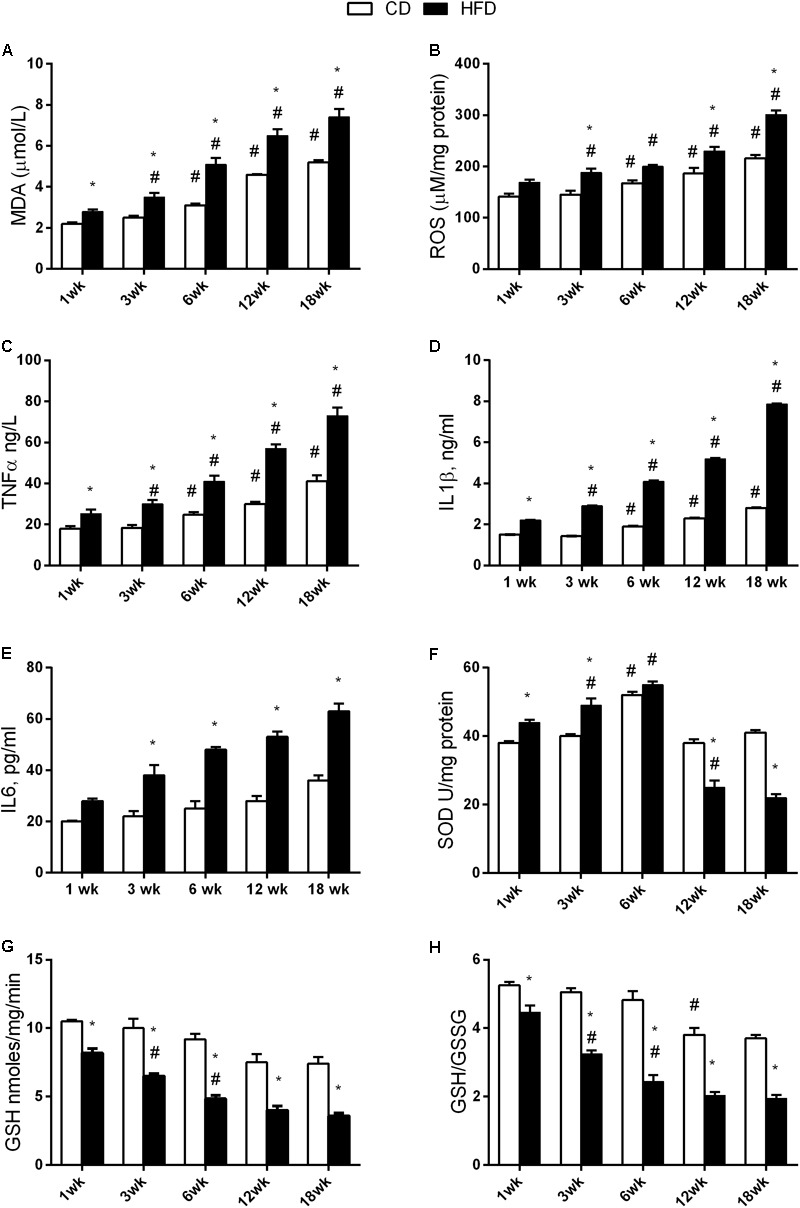
Effects of HFD and age on hypothalamic inflammation and oxidative stress. All the parameters were measured throughout the experimental period (1–18 weeks). MDA **(A)**, ROS **(B)**, TNF-α **(C)**, IL1β levels **(D)**, IL6 levels **(E)**, SOD activity **(F)**, GSH content **(G)**, and the GSH/GSSG ratio **(H)** are shown. Data are indicated as means ± SEM from *n* = 6 animals/group. ^#^Significantly different compared to previous age, *p* < 0.05; ^∗^significantly different compared to CD group, *p* < 0.05.

We observed a significant increase in MDA, ROS production, TNF-α, and IL1β with age in both the CD and the HFD group. This increase started at 6 weeks in CD group. The same increase was observed to occur earlier (at 3 weeks) in the HFD group. In addition, these values were significantly higher in the HFD when compared to the CD at each time point (**Figures 5A–D**).

The results of two-way ANOVA analysis for IL6 levels showed significant effect for the age [*F*(4,50) = 41.72; *p* < 0.01], diet [*F*(1,50) = 221.4; *p* < 0.01], and the interaction between the age and diet was not significant [*F*(4,50) = 6.532; *p* < 0.01].

IL6 levels did not change with age in both CD and HFD group, but they were significantly higher in the HFD group compared to CD, starting at 3 weeks of treatment (**Figure 5E**).

The results of two-way ANOVA analysis for SOD activity showed significant effect for the age [*F*(4,50) = 124.8; *p* < 0.01], diet [*F*(1,50) = 14.09; *p* < 0.01], and the interaction between the age and diet [*F*(4,50) = 55.43; *p* < 0.01].

SOD activity was higher at 6 weeks in the CD group when compared to any other age group. On the other hand, in the HFD group, SOD activity significantly increased at 3 and 6 weeks, and significantly decreased at 12 and 18 weeks (**Figure 5F**).

The results of two-way ANOVA analysis for GSH levels showed significant effect for the age [*F*(4,50) = 33.85; *p* < 0.01] and diet [*F*(1,50) = 191.8; *p* < 0.01], while the interaction between the age and diet was not significant [*F*(4,50) = 1.778; *p* > 0.05].

The results of two-way ANOVA analysis for GSH/ GSSG showed significant effect for the age [*F*(4,50) = 58.78; *p* < 0.01], diet [*F*(1,50) = 289.8; *p* < 0.01], and the interaction between the age and diet [*F*(4,50) = 6.681; *p* < 0.01].

A significant decrease in GSH (**Figure 5G**) and GSH/GSSG (**Figure 5H**) was observed at 12 weeks in the CD group. In the HFD group, this decrease was anticipated at 3 weeks. Such parameters were significantly different between the HFD and the CD group at every time point (**Figures 5G,H**).

The results of two-way ANOVA analysis for GSSG levels did not show significant effect for the age [*F*(4,50) = 0.2053; *p* > 0.05], diet [*F*(1,50) = 0.0575; *p* > 0.05], and the interaction between the age and diet [*F*(4,50) = 0.4343; *p* > 0.05].

The GSSG value did not change with age and diet (data not shown).

The results of two-way ANOVA analysis for GST activity showed significant effect for the age [*F*(4,50) = 5.925; *p* < 0.01], but did not show significant effect for the diet [*F*(1,50) = 1.23; *p* > 0.05] and interaction between the age and diet [*F*(4,50) = 0.0492; *p* > 0.05].

The results of two-way ANOVA analysis for NQO1 activity did not show significant effect for the age [*F*(4,50) = 1.813; *p* > 0.05], diet [*F*(1,50) = 0.7282; *p* > 0.05], and interaction between the age and diet [*F*(4,50) = 0.285; *p* > 0.05].

Moreover, no difference was detected in GST and NQO1 activity with age and diet (data not shown) suggesting that Nrf2 pathway is not involved in aging and HFD modulation of redox status.

### Effect of the HFD on the Phosphorylation of the Hypothalamic AMPK

**FIGURE 6:**
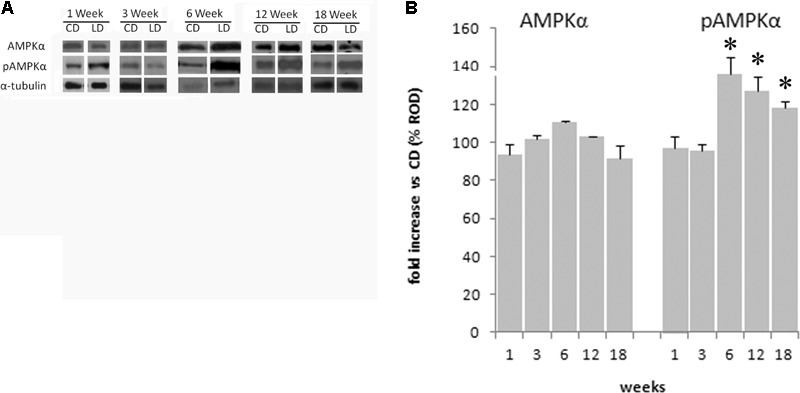
Effect of HFD and age on hypothalamic AMPK and pAMPK. **(A)** Representative blots probed with antibodies against p-AMPK (phosphorylated at Thr172), AMPK, and α-tubulin. Samples were obtained by hypothalamus of animals treated for 1–18 weeks with CD and HFD. **(B)** Quantitation of protein levels of AMPK and pAMPK normalized to the α-tubulin by densitometry. The bars show the values (means ± SEM) expressed as percentage of CD group as relative optical density (ROD). ^∗^Significantly different compared to CD group, *p* < 0.05.

The HFD did not modify the expression of AMPK in the hypothalamus at 1 (*p* = 0.30), 3 (*p* = 0.59), 6 (*p* = 0.25), 12 (*p* = 0.25), or 18 (*p* = 0.94) weeks, as shown by the results of Western blot analyses (**Figure 6**). To investigate the effects of the HFD on the activity of this protein, the expression level of pAMPK (the phosphorylated active form of AMPK) was analyzed. There was a significant increase of its level in the HFD compared to the CD group after 6 (*p* < 0.01), 12 (*p* < 0.05), and 18 (*p* < 0.05) weeks of treatment, while no significant differences were seen at 1 (*p* = 0.69) or 3 (*p* = 0.93) weeks of treatments (**Figure 6**).

## Discussion

Body composition is continuously changing with age (Iossa et al., 2000, 2002, 2003; Xu et al., 2005). Accordingly, our data showed a progressive increase in body fat mass with age. These changes are the result of diminished energy expenditure and increased metabolic efficiency. As a consequence, most of the energy intake is stored as fat. From a metabolic point of view, we observed an increase in lipidemic indexes and inflammation and a decrease in glucose tolerance. In the CD group, these changes started at about 6 weeks of treatments (about 100 days of age), and progressively increased with age. Interestingly, similar changes were observed in animals fed with HFD, but the variations were more pronounced and occurred earlier, at about 3 weeks of treatment (about 80 days of age). In particular, the body lipid increase in the HFD group is related to a decrease in energy expenditure and an increase in energy efficiency. The increase in energy efficiency, during high-fat feeding, may be partially due to the cost of lipid deposition, that in this conditions is lower than carbohydrates and proteins (Jéquier, 2002). In addition, during the high-fat feeding, the mismatch between fatty acid uptake and utilization leads to the accumulation of toxic lipid species resulting in overproduction of ROS, insulin resistance, and inflammation. Our results confirm the lipotoxicity of HFD. It is worth to notice that very young rats fed with HFD for 2 weeks exhibited decreased energetic efficiency due to the presence of adaptive thermogenesis, thus counteracting obesity development (Iossa et al., 2002). In addition, we showed that the body fat mass positively correlates with serum leptin levels and negatively correlates with adiponectin levels, thereby confirming previous results (Oda et al., 2008). Decreased adiponectin levels were demonstrated to be associated with decreased insulin sensitivity and to precede the onset of type 2 diabetes (Chakraborti, 2015). Adiponectin secretion is inhibited by several factors including high level of TNF-α and oxidative stress (Chakraborti, 2015). Therefore, our data, showing decreased adiponectin levels in the HFD group, associated with increased levels of TNF-α and oxidative stress, confirm the correlation between adiponectin levels and inflammatory state. The L/A ratio has been proposed as a better index of metabolic diseases, than independent levels of leptin and adiponectin, since it shows a better correlation to insulin resistance (Oda et al., 2008). Accordingly, our data indicated a higher L/A ratio in HFD rats, associated with higher HOMA index and inflammation.

Rat models of diet-induced obesity are characterized by inflammation both in peripheral tissues and in the hypothalamus, a brain region that plays a crucial role in energy homeostasis. Interestingly, unlike inflammation in peripheral tissues, which develops as a consequence of obesity, hypothalamic inflammatory signaling was evident in rodents within few days of HFD onset, prior to substantial weight gain. Therefore, the effects of HFD seem to start in the hypothalamus and affect peripheral tissues only after prolonged consumption of fat (Thaler et al., 2012). In fact, the HFD-induced inflammation, and the increase in oxidative stress in the hypothalamus was observed to start as early as after 1 week of treatment. The increased levels of ROS is well known to alter several cellular components as DNA, lipids, and proteins, thereby leading to neurons damage. These ROS can be scavenged by endogenous antioxidants. In particular, CuZn–SOD is the first line of defense against oxidative stress (Lu et al., 2009). Accordingly, our results indicated that CuZn–SOD activity is already elevated after 1 week of HFD and remained higher than CD group up to 3 weeks while markedly decreased after 12 weeks. The elevated SOD activity was able to balance ROS levels only for the first week. Indeed ROS levels increased progressively during the treatment reaching high values at 12 and 18 weeks when SOD activity was low. Since the molecular mechanisms linking the HFD administration to the inflammatory response at hypothalamic level is not completely known, we studied the effect of long-term high-fat feeding on the activation of AMPK in the hypothalamus. Hypothalamic AMPK, being able to control both feeding and energy expenditure, plays a key role in the modulation of whole-body energy homeostasis and body weight (López et al., 2016). We have recently shown that the administration of HFD to rats for 6 weeks does not modify the expression level of AMPK in the hypothalamus but significantly increases the level of the active phosphorylated form of the enzyme pAMPK (Viggiano et al., 2016). These results suggest that the increase in energy intake observed in HFD animals may depend on the activation of the hypothalamic AMPK pathway. Here, we reported that this key enzyme is still active after 12 and 18 weeks of HFD administration. This prolonged activation may be responsible, at least in part, of the increase in food intake observed in our animals. In addition, it has to be considered that activation of the hypothalamic AMPK is also responsible for alterations in fatty acid oxidation (He et al., 2006), leading to lipid overloaded hypertrophic adipocytes associated with inflammation and insulin resistance (Lionetti et al., 2009; Kim et al., 2015). Although the causal role of hypothalamic inflammation in HFD-induced obesity is well demonstrated (Thaler and Schwartz, 2010), the detailed molecular mechanisms coupling obesity-induced inflammation in hypothalamus and peripheral tissues are still uncertain. The earlier onset of inflammation in hypothalamus relative to peripheral tissues may play a crucial role in the establishment of response to chronic HFD exposure. Our data suggest that hypothalamic AMPK activation may mediate the interplay between hypothalamic and peripheral response to HFD.

## Conclusion

Our results, comparing standard diet and HFD in animals at the same age points, allow to define the kinetic of metabolic changes in the two groups, and indicates that the age at which HFD feeding starts, and the diet duration are both important factors in determining development of obesity. In addition, statistical analysis showed that the interaction between the age and HFD potentiates the negative effects on metabolic parameters. Moreover, in view of the importance of hypothalamic AMPK in controlling both feeding and energy expenditure, our data may be potentially useful in designing an innovative therapeutical approach for obesity and diabetes.

## Author Contributions

MPM and MC conceived and designed the experiments, analyzed the data, and wrote the manuscript. GC, EV, GT, CDF, AM, VM, AV, RC, CZ, FC, AC, and FS performed experiments, collected data, and performed data analyses. GM and MM contributed to the discussion and to the editing of the manuscript. MPM is the guarantor of this work and, as such, had full access to all the data in the study and takes responsibility for the integrity of the data and the accuracy of the data analysis.

## Conflict of Interest Statement

The authors declare that the research was conducted in the absence of any commercial or financial relationships that could be construed as a potential conflict of interest.
